# Efficacy of complete decongestive therapy (CDT) on edematous rat limb after lymphadenectomy demonstrated by real time lymphatic fluid tracing

**DOI:** 10.1186/2193-1801-2-225

**Published:** 2013-05-16

**Authors:** Yukari Takeno, Hiromi Arita, Etsuko Fujimoto

**Affiliations:** Graduate School of medicine, Nagoya University, 1-1-20 Daiko- Minami, Higashi-Ku, Nagoya-Shi, Aichi-Ken, 461-0047 Japan; Department of Nursing Science, Fukui Prefectural University, 4-1-1,Matsuoka-Kenjojima, Eiheiji-Machi, Yoshida-Gun, Fukui-Ken, 910-1195 Japan

## Abstract

Although complete decongestive therapy (CDT) is considered to reduce the volume of lymphedema, there is no concrete evidence to sustain its efficacy. The purpose of the present study was to find new evidence of CDT based on visualizing the changes of lymph fluid accumulating in an edematous limb using indocyanine green (ICG) fluorescent lymphography in real time.Twelve lymphedema rats were divided randomly into two groups. On the first day, ICG was injected into an edematous limb of rats, and no-intervention and CDT was applied to groups 1 and 2, respectively, for two weeks. ICG lymphography and circumferential measurements were done every two days in each two-week observation. The results indicates that a fluorescent flow to the ipsilateral axillary fossa was identified in all rats. In addition, network-like and dermal backflow patterns were observed in the lower legs and thighs. While manual lymph drainage was applied in the CDT group, the flow moved more rapidly through this pathway than that in the no-intervention group. An area of high-intensity fluorescent signals concentrated around the injection sites diminished in the CDT group more than that in the no-intervention-group after two weeks. Circumferential lengths of the edematous limbs were longer than the non-edematous limbs in both groups 1 and 2 on the day of ICG injection. The no-intervention group 1 showed no significance differences during 14 days, whereas the CDT group 2 exhibited very significant differences. These results suggest that CDT has beneficial effects in lymphedema treatment.

## Introduction

Lymphedema is a chronic inflammatory lymphostatic disease caused by mechanical failure of the lymphatic drainage system. According to a survey of characteristics of lymphedema in a developed country, the majority of the patients (80%) had cancer-related lymphedema, and 12% had non-cancer related lymphedema. Patients with primary lymphedema were only 8% (Sitzia et al. 1998). Especially, arm edema in women after axillary lymph node dissection for breast cancer is common (Szuba and Rockson 1998).

There are about 1.38 million new cases of breast cancer each year in major 184 countries (Ferlay et al. 2010). Although not all breast cancer patients who take surgery and/or radiation therapy develop secondary lymphedema, it is one of the most troublesome and chronic longterm aftereffects of damage to the lymph nodes (Loprinizi et al. 1999). Thus, the demand for lymphedema treatment is also expected to increase in the decade ahead.

Combined decongestive therapy (CDT) is an integral method of conservative treatment to ease lymphedema. Previous studies have tried to detect its efficacy in terms of the volume reduction of an edematous limb (Koul et al. 2007;Pinell et al. 2008) and an improvement in quality of life (Mondry et al. 2004). However, the International Society of Lymphology states that this therapy has yet to undergo a sufficient meta-analysis of multiple studies (International Society of Lymphology 2009). To prove this, CDT has been still uncovered by government insurance in many countries (Towers et al. 2008), and patients with lymphedema can difficultly seek it at low cost in public health service institutions. Therefore, a different approach to examine CDT is sought so that medical professionals can offer well-supported care to lymphedema patients.

Indocyanine green (ICG) lymphography has recently been used to treat lymphedema along with the development of a photodynamic eye system (PDE: Hamamatsu Photonics K.K. Hamamatsu, Japan) (Ogata et al. 2007;Unno et al. 2008). However, this novel method has yet to be used as an assessment tool to test the efficacy of CDT. Considering methodological difficulties and ethical issues to conduct experimental research using humans in lymphedema by ICG lymphography, the present study used animals because the models are easy to access, and also because it is possible to intervene during the study. The purpose of the present study was to find new evidence of CDT based on visualizing the changes of lymph fluid accumulating in an edematous limb using ICG lymphography.

## Materials and methods

### Materials

Twelve male SPF Wistar rats (age range, 9–10 weeks; Body weight 230~250 g) were used. The rats were purchased from Japan SLC Company (Hamamatsu, Shizuoka, Japan) and housed in groups of 2 per cage. Room temperature and humidity was maintained at 26°C and 40–60%, respectively, and automatic light control was set on 12:12-hr cycle. The rats could access to food and water with ad libitum.

All in vivo experimental protocol were reviewed and approved by the Animal Experiment Committee of Nagoya University.

### Surgical procedure

During surgery, rats were maintained with approximately 1.6% isoflurane. The hind limbs were shaved to measure a circumferential length at both groins. Then, 0.2 ml of a 100 mg/ml solution of Evans Blue (EB) dye was injected subcutaneously into the dorsum of the right paw and both the medial and the lateral ankles to detect lymph nodes (LNs) and vessels. An incision from a surface of the skin to subcutaneous tissue was made along the right inguinal ligament. Lymph nodes with surrounding adipose tissue in the inguinal site and in the ipsilateral popliteal fossa were excised. Dyed lymph vessels were carefully ligated under optical imaging by a 10–0 monofilament non-absorbed suture. Furthermore, a dyed LN in the pelvis was excised. The skin edges were sutured end to end by 4–0 nylon. No dressings or topical treatments were applied to any of the wounds. Analgesia and antibiotic was not given post operatively. Rats wore an Elizabethan collar for three weeks after surgery to prevent them from removing the sutures.

### Experimental schedule

**Figure 1:**
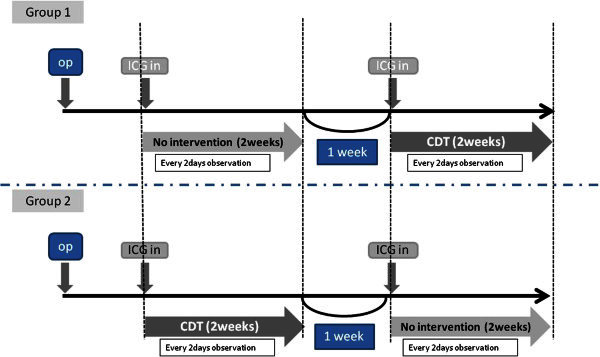
**Schedule of group 1 & 2.**

Five weeks after lymphadenectomy, all rats were divided randomly into two groups. An experimental protocol of both groups consisted of three parts: a two-week-observation, a one-week-interval, and a following two-week-observation (Figure 1). On the first day of each two-week observation period, 0.2 ml of a 1 mg/ml of ICG was injected into the same spots used when lymph nodes and vessels were dyed by EB. An observation by PDE was then performed under given certain condition.

Circumferential lengths were also measured at the proximal site of the incision line and at the untreated contralateral thigh bilaterally with a tape measure. ICG lymphography and circumferential measurements were done every two days in each two-week observation.

#### Group 1 (n=6)

No intervention was applied in the first two weeks. After the one-week interval, CDT was applied in the next two weeks.

#### Group 2 (n=6)

Combined decongestive therapy was initially applied in the first two weeks. After the one-week interval, no intervention was applied in the next two weeks.

### Procedure of CDT

Compression decongestive therapy consisted of manual lymph drainage (MLD), during which a compression bandage and exercise were used. Regarding MLD, only a stationary circle was performed for 15 minutes on the treated hind limb, the bilateral groins, and the right lateral side of the abdomen toward an ipsilateral axillary region from the right inguinal ligament. MLD was always done by the same person during the procedure. Bandage compression was applied using a cotton bandage for an under layer and a 1-cm–wide adhesive elastic bandage for overlapping layers proceeding in a proximal direction from the toe to the thigh. The rat with the compression bandage was released into a cage for two days. The rats could access to food and water with ad libitum.

### Data analysis

The images visualized by ICG lymphography were video-recorded and compared between the pre- and each observation period.

Bilateral differences in lengths were presented as mean ± standard divisions (SD). The significance of differences was statistically analyzed with repeated measures by ANOVA (analysis of variance) and by the Bonferroni multiple comparison test.

## Results

### Group 1

#### No intervention period

**Figure 2:**
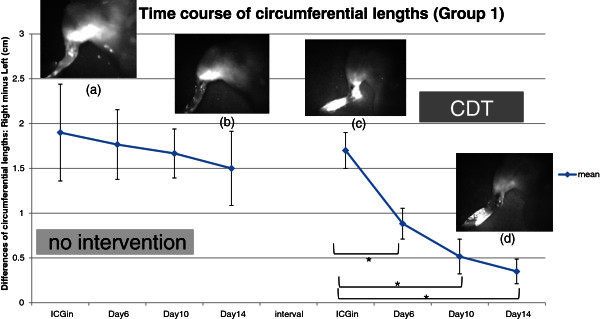
**The mean difference of circumferential lengths gradually decreases during the first 2-week period.** However, there are significant differences between the day of ICG injection and days 6, 10, and 14. Four photos are indicating the same hind limb. (**a**) is on the day of ICG injection, (**b**) is on day 14 of no intervention period. (**c**) and (**d**) are on the day of ICG injection and day 14 of CDT period, respectively.

On the day of ICG injection, a high-intensity fluorescent signal was concentrated around the injection sites (Figure 2a). In addition, network-like and dermal backflow patterns were identified in the lower legs and thighs 15 minutes after ICG injection. A fluorescent flow which moved from the lower abdomen toward the ipsilateral axillary fossa was observed in all the rats. Two of six rats had another route to pass transversely through the suprapubic region and then reach the contralateral inguinal fossa. A circumferential length of the treated hind limb was longer than that of the untreated limb in all rats. The mean ± SD between the limbs was 1.9 ± 0.54 centimeter (cm).

On day 2, the fluorescent pattern in the treated hind limb changed slightly compared to the day of ICG injection. The concentration of the fluorescent signal around the injection site was almost same as that on the day of ICG injection, except that the network-like pattern observed on the lower leg and thigh had disappeared. Instead, a diffuse-like fluorescence signal was distributed unevenly in the whole of the treated hind limb. No fluorescent flow to the ipsilateral axillary fossa was detected.

From days 4 to 14, fluorescent images were almost identical to those on day 2 (Figure 2b).

The mean ± SD in circumferential lengths between the limbs was reduced for 14 days to 1.5 ± 0.41 cm. There were no significant differences between the day of ICG injection and days 6, 10, and 14 (Figure 2).

#### CDT period

On the day of ICG injection, a high-intensity fluorescent signal was concentrated around the injection sites (Figure 2c). In addition, network-like and dermal backflow patterns were identified in the lower leg and thigh 15 minutes after ICG injection. A fluorescent flow to the ipsilateral axillary fossa was also observed in all the rats. Another route to the contralateral inguinal fossa was observed in two of them. While MLD was applied, the flow moved rapidly along this pathway. However, the area of the fluorescent signal in the treated hind limb did not noticeably change before and after MLD by PDE observation. The mean ± SD in circumferential lengths between the limbs was 1.7 ± 0.2 cm.

**Figure 3:**
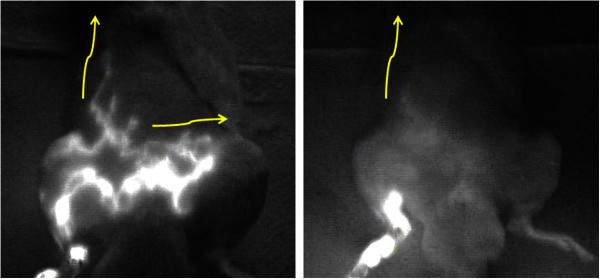
**Flourescent flows to the ipsilateral axillary fossa and the contralateral inguinal fossa are clearly observed (left).** On day 2, the route to the ipsilateral axillary fossa blurry but still observable after MLD are applied (right).

On day 2, the fluorescent pattern in the treated hind limb changed slightly compared to the day of ICG injection. The concentration of the fluorescent signal around the injection site was almost the same as on the day of ICG injection, but the network-like pattern observed on the lower leg and thigh had disappeared. Instead, a diffuse-like fluorescence signal was distributed unevenly in the whole of the treated hind limb. The route to the ipsilateral axillary fossa was blurry but still observable after MLD was applied (Figure 3).

Unlike the no intervention period, the fluorescent signal around the injection sites diminished gradually during the day from 4 to 14 (Figure 2d). On day 14, the mean ± SD among the limbs was 0.35 ± 0.14 cm. There were significant differences between the day of ICG injection and days 6, 10, and 14 (Figure 2).

### Group 2

#### CDT period

**Figure 4:**
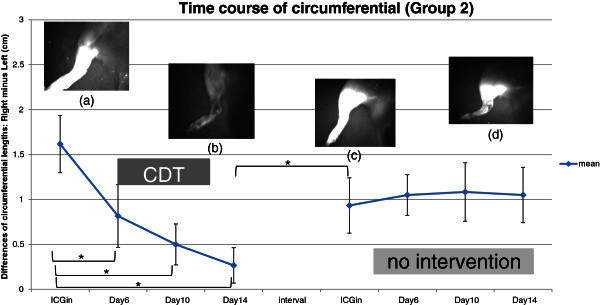
**The mean difference of circumferential lengths rapidly decreases during the first 2-week.** There are significant differences between the day of ICG injection and day 6, 10 and 14. However, there are no significant differences during the second 2-week. Four photos are indicating the same hind limb. (**a**) is on the day of ICG injection, (**b**) is on day 14 of CDT period. (**c**) and (**d**) are on the day of ICG injection and day 14 of no intervention period, respectively.

On the day of ICG injection, a concentration of the fluorescent signal around the injection sites was observed (Figure 4a). A high-intensity fluorescent signal was distributed from the foot pad to the ankle. In the lower leg and the thigh, network-like and dermal backflow patterns such as stardust were observed for least 15 minutes. A fluorescent flow that moved from the lower abdomen toward the ipsilateral axillary fossa appeared in all the rats. One of them had another route to the contralateral inguinal fossa. A circumferential length of the treated hind limb was notably longer than that of the untreated limb. The mean ± SD between the limbs was 1.62 ± 0.32 cm.

On day 2, the concentration of the signal around the injection site was almost the same as on the day of ICG injection, but the dermal backflow patterns on the lower leg and thigh had disappeared. Instead, a fluorescent signal was distributed diffusely and unevenly in the whole of the treated hind limb. The shape of the route to the ipsilateral axillary fossa was ambiguous (Figure 3).

On day 14, the fluorescent signal around the injection sites had decreased in comparison with day 2 (Figure 4b). The mean ± SD in the lengths also diminished to 0.27 ± 0.2 cm. There were significant differences between the day of ICG injection and days 6, 10, and 14 (Figure 4).

#### Interval

A difference in circumferential lengths increased during one week. After a *t*-test, the difference was significant one (Figure 4).

#### No intervention period

On the day of the ICG injection, a concentration of the fluorescent signal around the injection sites was observed (Figure 4c). A high-intensity fluorescent signal was distributed from the foot pad to the ankle. In the lower leg and thigh, network-like and dermal backflow patterns such as stardust were observed for least 15 minutes. A fluorescent flow which moved from the lower abdomen toward the ipsilateral axillary fossa appeared in all rats. A circumferential length of the treated hind limb was longer than that of the untreated limb. The mean ± SD between the limbs was 0.93 ± 0.31 cm.

The concentration of the fluorescent signal around the injection sites changed little for 14 days in comparison to that in the CDT period (Figure 4d). The mean ± SD in lengths between the limbs increased slightly to 1.05 ± 0.31 cm in comparison with the day of the ICG injection. However, there were no significant differences between the day of the ICG injection and days 6, 10, and 14 (Figure 4).

### Comparison between groups 1 and 2

During the first two-week period of both groups, no intervention for group 1 and CDT for group 2 were compared.

The fluorescent images observed on the day of ICG injection were almost identical between groups. The fluorescent flow to the ipsilateral axillary fossa was identified in all rats. However, while MLD was applied in group 2, the flow moved more rapidly through this pathway than that in group 1.

The fluorescent signal concentrated around the injection sites decreased for 14 days in groups 1 and 2. However, a weaker signal was detected in the CDT group (Figure 4a, b) than that in the no-intervention group (Figure 2a, b).

Circumferential lengths of the treated hind limb were longer than untreated limbs in both groups 1 and 2 on the day of ICG injection. Group 1 (no intervention) showed no significance differences during 14 days, whereas group 2 (CDT) exhibited significant differences.

## Discussion

The fluorescent signal presented with a network-like pattern in the treated hind limb travelled beyond the incision line, where it became a linear pattern at the right lateral region of the abdomen reaching to the right axilla. The network-like and linear patterns represent lymph capillaries and collectors, respectively. Therefore, lymph fluid that drained from the periphery by CDT moved through lymph capillaries, flowed into lymph collectors, and then reached the axillary LN. A reduction of an edematous limb by an application of CDT has been well-documented (Koul et al. 2007;Pinell et al. 2008). However, pathways of lymph fluid and destination LNs have never been studied scientifically. The present study revealed drainage routes of lymph fluid in a lower limb lymphedema.

The PDE system captured the high-intensity area of a fluorescent signal around the injection site after ICG was injected into the subcutaneous space of the right paw and ankle. After CDT was applied for two weeks, the high-intensity area at the injection site was reduced, and the low-intensity fluorescent signal was increased instead. These changes indicated that the application of CDT to the edematous limb encourages lymph fluid to move from the periphery. This is the first demonstration that the efficacy of CDT has been visualized. ICG is less invasive, and its fluorescent navigation is very sensitive. The detection rates of sentinel LNs and lymphatic channels are 94~97% (Hirche et al. 2010;Kitai et al. 2005) and 100% (Abe et al. 2011;Kitai et al. 2005), respectively.

In both groups, the high-intensity area of the fluorescent signal was reduced after the 2-week-CDT, but there was little reduction without CDT for another 2 weeks. The changes in fluorescent images were well-supported by the changes in circumferential lengths, thus rendering the evidence becomes clearer than ever before. Each assessment method has several weak points. The present study offers a new experimental method that compensates for the weaknesses of CDT research.

The efficacy of CDT was studied using lymphedema rats in the present study. This experimental model is far only for early edema but not acute postoperative edema. Although the swelling occurs after surgical invasion in the acute period, it improves over several days after surgery. Rats used in the present study showed images indicated as lymphedema by ICG lymphography at the beginning of the experimental schedule between five and seven weeks after lymphadenectomy. This may show sustained lymphedema after acute postoperative edema disappear.
